# Recent Advances in Microelectrode Array Interfaces for Organoids

**DOI:** 10.3390/biomimetics11020142

**Published:** 2026-02-13

**Authors:** Dongha Kim, Hanjun Ryu

**Affiliations:** 1 Department of Intelligence Energy and Industry, Chung-Ang University, Seoul 06974, Republic of Korea; rlaehdgk12@cau.ac.kr; 2 Department of Advanced Materials Engineering, Chung-Ang University, Anseong-si 17546, Republic of Korea

**Keywords:** microelectrode array, organoid, tissue interface, 3D structure, stem cell

## Abstract

Electrophysiological studies using brain organoids provide valuable insights into neurological disorders and offer promising opportunities for therapeutic development. Accordingly, conventional two-dimensional microelectrode arrays (MEAs) are commonly employed to record neural activity with high spatiotemporal resolution. However, their measurements are mainly limited to the basal surface of the tissue. This limitation restricts the comprehensive analysis of the complex three-dimensional (3D) neural networks formed within organoids. To bridge this gap, this review summarizes recent advances in 3D MEA technologies, with a focus on device geometries, electrode designs, and neural signal acquisition strategies ranging from noninvasive to invasive approaches. Among these advances, photolithography-based fabrication processes have enabled submicron-scale structures, improving device flexibility, spatial resolution, and signal-to-noise ratio. Furthermore, the integration of 3D MEAs with perfusion systems and shape-transformable architectures facilitates stable, long-term electrophysiological monitoring of organoids. Finally, this review discusses emerging research trends and future perspectives in 3D MEA development in organoid-based neuroscience.

## 1. Introduction

Direct experimental access to the human brain is inherently limited, posing challenges for the study of human brain development and neurological disorders. To address these limitations, three-dimensional (3D) neural culture systems derived from human stem cells (i.e., organoids) have garnered increasing attention [1]. Unlike conventional two-dimensional (2D) cell cultures, brain organoids formed through three-dimensional neural differentiation exhibit advanced cellular composition, maturation, and overall tissue architecture that more closely recapitulates the human brain [2,3]. Consequently, brain organoids have been proposed as models for investigating the mechanisms of human brain development and neurological diseases, as well as for establishing a foundation for therapeutic studies [4,5].

Neuronal communication occurs through synaptic transmission, in which neurotransmitters are released and conveyed to postsynaptic neurons, eliciting postsynaptic potentials. Techniques that measure intracellular membrane potentials and extracellular electrical signals enable the detection of neuronal communication and facilitate the analysis of neural network activity in biological systems, thereby advancing the understanding of nervous system function. Such techniques provide critical insights into the pathophysiology of various neurological disorders [6,7]. Patch-clamp recording is the most widely used method for directly measuring intracellular membrane potentials at the single-cell level, offering high temporal resolution and precise characterization of neuronal signals. Its ability to resolve ion channel responses makes it particularly useful for evaluating pharmacological effects [8,9]. However, this method is limited to individual cells, restricting its use in analyzing network connectivity and dynamics in 3D tissues such as organoids. Calcium imaging partially overcomes this limitation by enabling real-time visualization of neuronal activity in small populations of neurons and allowing region-specific analyses [10,11]. Nevertheless, calcium imaging does not directly measure electrical signals and remains limited in its ability to resolve neuronal connectivity and network-scale dynamics in 3D environments. Moreover, it may induce phototoxic effects during repeated optical excitation, rendering it unsuitable for long-term measurements. To circumvent these challenges, MEAs have emerged as a prominent electrophysiological platform that enables a noninvasive and stable means of recording extracellular potentials [12].

MEAs enable simultaneous recording and stimulation of multiple cells through microscale electrodes and can reliably detect action potentials (APs) generated during neuronal firing as well as local field potentials resulting from the summed synaptic activity of large neuronal populations [13]. MEA systems can be broadly classified into passive and active electrodes. Passive MEA electrodes route signals to external amplifiers through metal interconnects, whereas active MEA electrodes incorporate complementary metal–oxide–semiconductor (CMOS) technology to integrate amplifiers and analog-to-digital converters directly onto the same substrate as the electrodes. These MEA systems enable long-term analysis of functional connectivity and activity patterns at the neural network level, establishing MEAs as core tools for brain organoid studies [14,15]. Capturing these electrophysiological signals with high fidelity requires both high temporal resolution and a high signal-to-noise ratio (SNR). However, as electrode density increases, the electrode area decreases, leading to increased impedance and a corresponding decrease in SNR. To mitigate this trade-off, surface coatings such as platinum black and PEDOT:PSS are commonly applied to MEAs to increase the effective electrode surface area and thus improve the SNR. In addition to enabling electrophysiological recording, MEAs support on-device cell culture and electrical stimulation, making them particularly well suited for 3D biological systems such as organoids. Electrical stimulation during the early stages of organoid culture has been reported to enhance organoid development and maturation, further highlighting the advantages of MEA platforms for 3D tissue culture and analysis [16,17].

Thus, this review focuses on MEA systems developed for the electrophysiological study of neural tissues. Conventional planar MEAs are not suitable for capturing the 3D organization of biological systems such as organoids, as electrode–tissue contact is largely confined to the basal surface, thereby limiting functional analysis. To address these limitations, MEAs have been explored in a wide range of architectures, ranging from conventional planar configurations to 3D designs and CMOS-integrated systems. These 3D MEA platforms reflect the structural characteristics of organoids while enabling functional electrophysiological interrogation. This review compares and summarizes diverse MEA platforms for brain organoids in terms of geometry, materials, recording performance, and organoid compatibility. Based on these aspects, it aims to provide a useful framework to guide the design and selection of MEA platforms for specific applications, such as studies of neural development, disease modeling, and drug screening.

## 2. MEA Architectures

### 2.1. High-Density CMOS MEAs for Single-Unit Analysis in Brain Organoids

**Figure 1:**
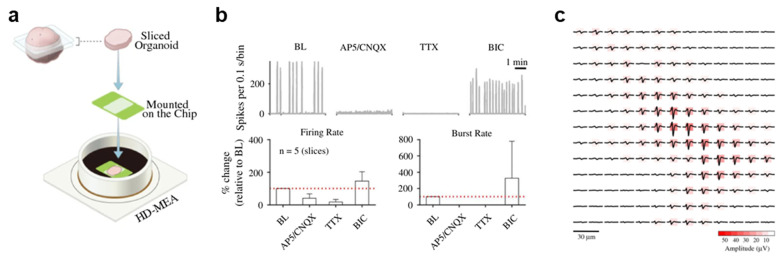
**HD-MEAs.** (**a**) Schematic illustration of sliced human cerebral organoids cultured on an HD-MEAs. (**b**) Effects of glutamatergic receptor antagonists (AP5 and CNQX) and a sodium-channel blocker (TTX) on spontaneous activity in human cerebral organoids. Baseline (BL) activity, quantified by summing activity across all channels at 0.1 s intervals, decreases upon application of CNQX and AP5 and is completely suppressed after the addition of TTX. Application of the GABA_A receptor blocker bicuculline (BIC) increases overall activity and burst frequency. (**c**) Electrical footprints of human cerebral organoids neurons recorded using an HD-MEAs. Reproduced with permission from ref. [19]. Copyright © 2022, The Authors.

Human brain organoids are considered suitable model systems for investigating key physiological mechanisms underlying early neuronal network formation [1,18]. However, conventional MEAs, which typically feature few electrodes (<100 electrodes per mm^2^) and wide interelectrode spacing, are limited to evaluating only population-level activity. In contrast, CMOS-based high-density MEAs (HD-MEAs) offer high spatiotemporal resolution, enabling efficient separation of neuronal signals and facilitating single-unit analysis [19]. A representative HD-MEA comprises 26,400 platinum microelectrodes (9.3 × 5.3 μm^2^) arranged at a 17.5 μm pitch, covering a total sensing area of 3.85 × 2.10 mm^2^. This architecture supports simultaneous recording from up to 1024 electrode channels at a 20 kHz sampling rate. To optimize signal quality, the electrodes are coated with platinum black, which decreases the impedance to 1–10 kΩ at 1 kHz [20] and increases the SNR (2.4 µV_rms_ in the 300 Hz–10 kHz range) [21]. In a previous study, spontaneous neural activity was recorded from 100-day-old mature brain organoids derived from human-induced pluripotent stem cells (hiPSCs) and human embryonic stem cells. The organoids were sectioned into slices and placed on HD-MEAs (Figure 1a), and robust network bursts were detected in 12 out of 14 slices. These organoid slices exhibited a burst rate of 0.01 ± 0.01 Hz, a mean burst duration of 4.05 ± 1.18 s, and a mean firing rate of 0.38 ± 0.80 Hz, reflecting their spontaneous responses. The application of 6-cyano-7-nitroquinoxaline-2,3-dione (CNQX) and D-(-)-2-amino-5-phosphonopentanoic acid (AP5) receptor antagonists, which target α-amino-3-hydroxy-5-methyl-4-isoxazole-propionic-acid (AMPA) and N-methyl-D-aspartate (NMDA) receptors, respectively, reduced the recorded activity to ~40% of the baseline; conversely, the sodium-channel blocker tetrodotoxin (TTX) further suppressed it to 16% (Figure 1b). In contrast, the GABA_A receptor antagonist bicuculline moderately increased neural activity. These pharmacological responses indicate that the observed network dynamics are driven by interactions between excitatory and inhibitory neurons. HD-MEAs allow for the detection of electrical footprints, which occur when a single neuron generates APs waveforms that are simultaneously detected across multiple adjacent electrodes (Figure 1c). By extracting the signal from the electrode exhibiting the highest amplitude within each electrical footprint and applying waveform clustering using the Louvain community detection algorithm, signals from the soma or axon can be distinguished. In the study, HD-MEAs consisting of a 20 × 20 electrode array with a 17.5 μm pitch were employed to compute axonal AP propagation velocities. The conduction speed was estimated based on the delay in AP timing across electrodes as a function of distance along the axon. An analysis of 339 units yielded a mean axonal conduction velocity of 0.41 ± 0.15 m/s, which is consistent with reported values for hiPSC-derived neurons. The mean tracking distance for axonal APs on the HD-MEAs was 111 ± 50 μm, representing a noninvasive measurement of axonal conduction properties in human brain organoids. Overall, HD-MEAs enable large-scale functional analysis of human brain organoid activity at both the cellular and network levels, supporting the physiological relevance of organoids as models of the human brain and highlighting their utility for disease modeling and drug screening [22,23].

### 2.2. Mesh MEAs for Long-Term Recording of Organoids

**Figure 2:**
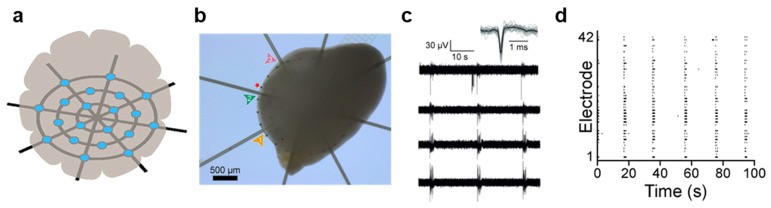
**Mesh MEAs.** (**a**) Schematic illustration of the mesh MEA. Blue circles indicate microelectrode position, and black lines indicate substrate. (**b**) Optical microscopy image of an organoid growing on the mesh MEA. Colored arrows indicate substrate and red star indicates microelectrode. (**c**) Spontaneous activity of the organoid recorded from four electrodes, with the AP waveforms exhibiting peak amplitudes of up to ~50 μV. (**d**) Synchronized bursting activity detected across 42 microelectrodes of the mesh MEA, shown as a spike raster plot. Reproduced with permission from ref. [25]. Copyright © 2023 Elsevier B.V.

Conventional 2D MEAs and invasive electrodes inadequately capture the 3D architecture of neural organoids, often impeding growth and causing tissue damage, which limits their suitability for long-term recordings [23,24]. In contrast, 2D mesh structures form a 3D interface that alleviates the compressive effects inherent to planar MEAs, supporting natural tissue development and addressing these limitations. For example, a hammock-like mesh MEA comprising 61 electrodes suspends hiPSC-derived neural organoids, allowing them to grow unrestrictedly and envelop the mesh (Figure 2a,b) [25]. This configuration minimizes interference with the network activity of structurally developing organoids and enables stable, long-duration electrophysiological recordings. The device features a spiderweb-like mesh suspended 2 mm above a glass substrate. The mesh consists of a 12 μm-thick polyimide layer patterned with titanium nitride microelectrodes with a diameter of 30 μm and spacing of 200 μm. The titanium nitride electrodes exhibit impedance magnitudes below 100 kΩ at 1 kHz, rendering them suitable for electrical stimulation and recording. During testing, hiPSC-derived neural organoids were allowed to grow freely for 197 days before being cultured on the mesh MEA for an additional year. The 12 µm-thick mesh occupied approximately 0.1% of the volume of a 2 mm-diameter organoid, allowing unconstrained growth in all directions without limiting oxygen supply. After 35 days on the mesh, spontaneous activity—including spikes up to 50 μV and synchronized bursting—was detected from 69% of the electrodes (42 out of 61) (Figure 2c,d). Notably, no structural damage to the organoids was observed even after one year of culture on the device. Collectively, these findings demonstrate the mesh MEAs are suitable for long-term organoid culture and noninvasive electrophysiological monitoring of developing neural tissues.

### 2.3. Self-Folding Shell MEAs for Encapsulating Organoids

**Figure 3:**
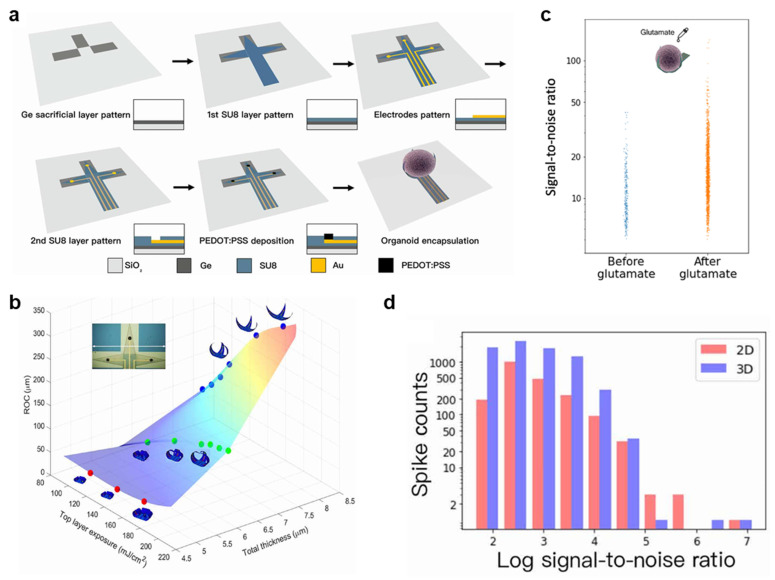
**Shell MEAs.** (**a**) Fabrication process of the shell MEA. (**b**) Simulated radius of curvature (ROC) plotted against SU-8 bilayer thickness and upper-layer exposure energy. (**c**) Comparison of spike distributions before and after glutamate application. (**d**) Spike counts with different SNRs recorded on the 2D and 3D platforms, revealing higher spike detection on the 3D platform. Reproduced with permission from ref. [27]. Copyright © 2022 The American Association for the Advancement of Science.

Planar MEAs are limited in their ability to record organoid electrophysiology because they inadequately capture the 3D architecture of organoids and restrict the electrode–tissue interface to the basal surface [26]. To address these limitations, 3D shell MEAs, inspired by electroencephalography caps, have been developed to envelop organoids and provide a substantially larger recording interface than conventional MEAs [27]. For example, self-folding polymer bilayers have been used to fabricate customized shell MEAs that accommodate organoids of varying sizes, enabling either loose or tight contact between the electrodes and the tissue (Figure 3a). The shell MEA consists of three leaflets that fold to encapsulate the organoid, with one electrode positioned on each leaflet at a lateral interelectrode spacing of 1450 μm. During fabrication, photolithography was used to pattern a 50 nm Au electrode layer beneath a self-folding SU-8 bilayer (8.0 μm, 6.0 μm, or 4.6 μm). The folding behavior of the bilayer can be tuned by adjusting the thickness and UV exposure energy. Differential UV exposure of the upper and lower SU-8 layers creates a gradient in cross-link density, causing the two layers to swell to different extents during solvent exchange from acetone to water or culture medium, thereby inducing self-folding. By varying the upper-layer thickness and UV exposure levels (4.6 μm at 180 mJ/cm^2^, 6.0 μm at 180 mJ/cm^2^, and 8.0 μm at 120 mJ/cm^2^) and fully cross-linking the lower layer (at 240 mJ/cm^2^), it was discovered that thin bilayers and large UV exposure differentials resulted in greater folding curvature (Figure 3b). These tunable parameters enabled the encapsulation of organoids with diameters of 400–600 μm, demonstrating the feasibility of customized shell fabrication. To improve recording quality, a 10 μm layer of the conductive polymer PEDOT:PSS was deposited onto the electrode surfaces, reducing impedance from >100 kΩ to <10 kΩ at 1 kHz and enhancing electrode–organoid interfacing. Brain organoids were generated using the iPSC line NIBSC8. The 3D MEA successfully recorded spontaneous activity with amplitudes up to 200 μV. Furthermore, the application of 20 μM glutamate significantly increased the spike SNR by a median of 57.6% (Figure 3c). Since the self-folded 3D MEA positions its electrodes close to the organoid, it was able to detect 7785 spikes, outperforming planar 2D electrodes, which detected 2025 spikes. Moreover, the same spikes exhibited higher SNRs in the 3D electrode channels than in the 2D channels, with a median increase of 42% (Figure 3d). These findings demonstrate that 3D bioelectronic platforms provide superior electrophysiological signal capture compared to conventional 2D electrodes, highlighting their potential for spatially resolved neural analysis. Thus, shell MEAs enable customized designs for organoids of varying sizes and provide 3D recording capabilities, opening opportunities for detailed functional characterization of brain organoids.

### 2.4. Kirigami MEAs for Self-Transforming 3D Interfacing with Suspended Organoids

**Figure 4:**
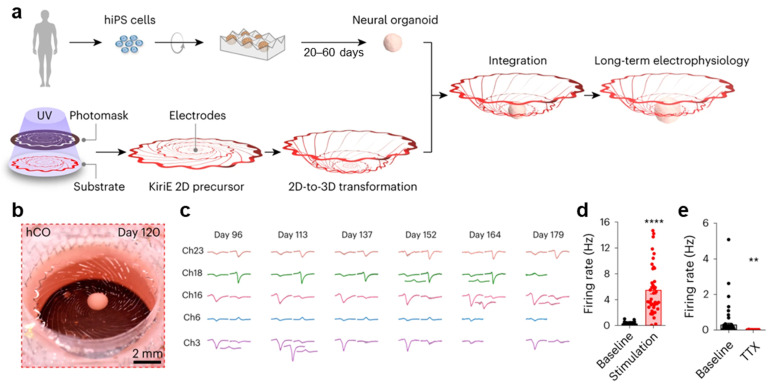
**Kirigami MEAs.** (**a**) Schematic illustration of the integration of a neural organoid with a deformable KiriE platform. (**b**) Image of an hCO cultured on the KiriE on day 120 of differentiation. (**c**) Representative spike waveforms recorded from five channels on days 96, 113, 137, 152, 164, and 179 of differentiation. Scale bars: 100 µV (vertical) and 1 ms (horizontal). (**d**) Firing rates of two hCOs with and without optical stimulation. (**e**) Firing rates recorded after TTX treatment. Reproduced with permission from ref. [28]. ** *p* < 0.01. **** *p* < 0.0001. Copyright © 2024, The Authors.

Patch-clamp techniques and planar MEA platforms are poorly suited for long-term electrophysiological recordings because they disrupt the fully suspended 3D self-organization of neural organoids. Kirigami electronics (KiriEs), which leverage geometric cutting and out-of-plane folding, are engineered to accommodate freely floating neural organoids, thereby enabling stable, long-duration recordings [28]. KiriEs transform from a planar kirigami pattern into a self-folded, 3D basket-like structure (diameter: 1 cm) that encloses a human cortical organoid (hCO) once the organoid is placed at the center of the suspended 2D sheet (Figure 4a,b). The device contains 32 microelectrodes, each 25 μm in diameter, arranged within a 1 mm central region and exhibiting an impedance of approximately 300 kΩ. The KiriE pattern has a total thickness of about 0.9 μm, which includes the thickness of the SU-8 insulating layer for the embedded metal traces. During fabrication, two geometries—spiral and honeycomb—were designed to ensure mechanical robustness and sufficient deformability to withstand potential stresses. The spiral pattern consists of interconnected concentric rings joined by spiral latches, which rotate and expand vertically under load. Finite element method simulations were employed to modify the spiral design by rounding sharp corners to prevent strain accumulation and introducing wavy concentric rings to reduce in-plane tension. Among 150 simulated geometries, the spiral latch design was optimized for smooth deformation, minimizing localized strain while allowing adequate vertical extension. The selected latch length maintained structural stability, limiting the maximum strain to 0.06%, well below the elastic limit of SU-8 (2–4%), even when supporting organoids with diameters of 0.2–1.2 mm, as well as the weight of the KiriE. Moreover, hCOs derived from hiPSCs were placed onto the KiriE devices on day 20 of differentiation and monitored after integration. Unlike hCOs plated on adhesive substrates, which flattened within 14 days, the hCOs maintained a spherical morphology for at least 60 days on the KiriE device. SYTOX staining and cleaved caspase-3 (c-Cas3) expression assays revealed that the hCOs cultured on adhesive substrates exhibited higher levels of cell death, while the suspended organoids exhibited improved viability. Spontaneous neural activity was detected after day 96 (Figure 4c), with spike waveforms of 30–140 μV remaining stable over several days. Following transduction of the hCOs with adeno-associated virus (AAV), optical stimulation was applied from the bottom of the chamber. Quantification of firing rates demonstrated that activity during light-on periods was approximately 20-fold higher than that during light-off periods. The application of the potassium-channel blocker 4-aminopyridine (4-AP) increased the firing frequency from 0.49 ± 0.08 Hz to 1.60 ± 0.25 Hz, whereas TTX abolished spontaneous activity, demonstrating that KiriEs reliably capture neural responses (Figure 4d,e). In hCOs derived from hiPSCs carrying a heterozygous loss-of-function mutation in *DGCR8*, spontaneous firing rates differed by a factor of three compared to those of control hCOs on days 137–138, indicating that KiriEs can detect disease-related phenotypes. Additionally, the fusion of hCOs with human striatal organoids (hStrOs) produced assembloids, and the KiriE enabled stable interfacing with the integrated neural structures. Thus, the KiriE platform supports long-term spherical maintenance and recording of organoids for up to 179 days without insertion or substrate contact while enabling optical stimulation, pharmacological perturbation, and genetic interrogation. These capabilities highlight its potential for developing organoid-tailored neuroengineering systems.

### 2.5. Multifunctional 3D MEAs for Neural Circuit Analysis

**Figure 5:**
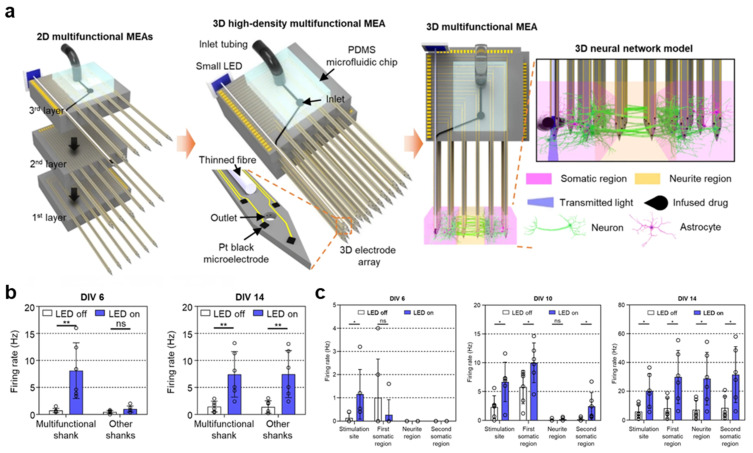
**Multifunctional 3D MEAs.** (**a**) Photograph of a multifunctional 2D MEA and the assembled 3D MEA, along with a schematic illustration of its application to a 3D neural network model. (**b**) Firing rates recorded from the multifunctional shank and other shanks during optical stimulation. (**c**) Firing rates recorded from the first somatic, neurite, and second somatic regions during optical stimulation. Reproduced with permission from ref. [32]. * *p* < 0.05. ** *p* < 0.01. Copyright © 2021, the authors.

Calcium imaging and planar 2D MEAs have a limited ability to analyze neuronal connectivity and network dynamics within 3D microenvironments, and existing 3D MEAs suffer from low electrode density and randomly distributed recording sites, making it difficult to monitor neural circuits [29,30,31]. Nevertheless, 3D multifunctional MEA systems overcome these limitations by integrating high-density electrodes, precise electrical stimulation control, and localized optical and pharmacological delivery, thus enabling the measurement of synaptic delays and transmission velocities within 3D neural tissues [32]. These systems are constructed by assembling three layers of 2D multifunctional MEAs (Figure 5a), each containing six shanks. This results in a 3 × 6 shank array, where one shank is engineered as a multifunctional unit that incorporates a thin optical fiber (diameter: 60 μm) and a microfluidic channel, whereas the remaining 17 shanks function as recording shanks. Each shank is 6 mm in length and integrates 63 Pt microelectrodes (20 × 20 μm^2^) at the tips. The application of the platinum black coating reduces the electrode impedance from 1.761 ± 0.346 MΩ to 15 ± 4 kΩ at 1 kHz, enhancing the neural-recording performance. Localized drug delivery is enabled by integrating a polydimethylsiloxane (PDMS) microfluidic chip with the 3D MEA, while optical stimulation is enabled by attaching a miniature LED to the end of the optical fiber. For demonstration, primary cortical neurons from Sprague Dawley (SD) rat embryos (E18) were seeded in collagen to create a single-group neural network and a compartmentalized two-group network. In the single-group model, neural activity was monitored over 14 days. Spontaneous firing first appeared on several electrodes by day in vitro (DIV) 6, and both burst frequency and the proportion of spikes within bursts increased markedly after DIV10. Quantification of interelectrode synchrony revealed progressive network synchronization, with the number of synchronized electrodes increasing over time. To verify functional activity, the neurons were then transduced with an AAV-EF1α-ChR2-eGFP virus to express the light-sensitive ion channel ChR2 and subsequently subjected to optical stimulation (0.2 Hz, 50% duty cycle, 76 mW/mm^2^). Under identical stimulation, firing at DIV6 was restricted to electrodes near the stimulated shank; however, by DIV14, optical stimulation evoked firing across the entire neural tissue (Figure 5b). After the infusion of the excitatory synaptic transmission blockers CNQX (20 μM, 1 μL) and AP5 (50 μM) at a flow rate of 0.25 μL/min for 4 min, subsequent optical stimulation elicited firing only near the stimulation site. This indicates that optically evoked activity propagates through excitatory synaptic transmission across the network. In the two-group network model, the multifunctional shank was positioned exclusively within the first somatic region, where optical stimulation was applied. At DIV6, neural activity was confined to electrodes near the first somatic region. By DIV9–10, firing rates increased in the second somatic region, and by DIV14, elevated firing rates and an increased number of active networks were observed across all regions (Figure 5c). These results confirm that APs propagate between distinct neuronal populations. Accordingly, performing optical stimulation and aligning spike timing across all electrodes to the stimulation onset allow for the estimation of signal propagation velocity. Neural activity was further evaluated in a hiPSC-derived spinal cord organoid with a diameter of ~700 μm using a needle-type MEA integrating 16 electrodes and a microfluidic channel. Neural activity was successfully recorded on all 16 electrodes, and TTX application resulted in an abrupt cessation of activity. This observation indicates that 3D MEAs can be applied to organoid systems to monitor neural activity within the tissue interior. Overall, 3D multifunctional MEA systems can enable dynamic analysis of neural circuits in organoids, serving as versatile platforms for investigating brain function and neurological diseases.

## 3. Conclusions

This review summarizes diverse MEA platforms designed to record electrophysiological signals while accounting for the structural characteristics of brain organoids, with quantitative platform specifications compared (Table 1) and the main strengths and limitations of each platform summarized (Table 2). Conventional 2D interfaces do not readily accommodate the inherently three-dimensional architecture of brain organoids or the morphological changes that occur during prolonged culture, making it challenging to consistently ensure stable electrode–tissue contact and reliable measurements. By contrast, MEAs with 3D architectures can conform to organoid morphology and maintain stable electrode–tissue interfaces, thereby further underscoring the need for 3D MEA approaches. Furthermore, MEA systems that integrate optical stimulation, pharmacological delivery, and high-density electrodes extend beyond simple neuronal recording and enable direct analysis of neural network-level dynamics, demonstrating their applicability for investigating brain function and neurological disorders. In particular, integrating patient derived brain organoids with MEA platforms may provide a preclinical tool to assess functional drug responses in a patient specific manner, thereby offering the potential to support truly personalized treatment strategies. However, challenges remain, including the trade-off between electrode density and recording depth, the establishment of stable long-term neural interfaces, and the need to establish standardized organoid specifications compatible with MEA interfacing, because variability in organoid size and morphology can hinder consistent electrode–tissue contact and recording quality, thereby limiting robust quantitative and qualitative MEA-based analyses. Also, to strengthen the clinical potential of organoid–MEA platforms, systematic biological validation, benchmarking against animal models, and the accumulation of clinical data should be addressed as important directions in future studies. To address these challenges, recent efforts have focused on interface designs that adapt to organoid growth and morphological changes to reduce mechanical mismatch and tissue damage [33], as well as on approaches that integrate optical analysis modalities with MEA-based electrophysiology to capture neural activity with high spatial and temporal resolution, thereby enabling multimodal electrical–optical interrogation of brain organoids [34,35]. In addition, further studies are required to improve SNRs through the reliable recording of extracellular APs, to accommodate morphological changes during prolonged organoid culture on MEA platforms, and to develop new methodologies for high-throughput evaluation of organoids. In this context, machine and deep learning-based frameworks for automated signal processing, feature extraction, classification, and quality control of large-scale 3D MEA datasets represent an important direction for advancing efficient and reproducible electrophysiological analyses [36,37]. Collectively, these advances in 3D MEA technologies are expected to enable detailed analyses of organoid-based neural circuit dynamics and to support future research into nervous system functions and diseases, as well as pharmacological screening aimed at optimizing patient-specific therapeutic strategies.

## Figures and Tables

**Table 1:** Summary of MEA applications for neural systems.

Electrode Type	Cell Type	Electrode Area (μm^2^)	Number of Electrodes	Electrode Impedance (at 1 kHz)	Signal Voltage (µV)	Ref.
Active CMOS	iPSCs and ESCs	49.29	26,400	1–10 kΩ	20–60	[19]
Passive 2D planar	iPSCs	702	61	100 kΩ	50	[25]
Passive 3D	iPSCs and ESCs	-	3	10 kΩ	200	[27]
	iPSCs	490	32	300 kΩ	30–140	[28]
	Rat cortical tissue	400	63	15 kΩ	100	[32]

**Table 2:** Summary of data processing approaches, strengths, and limitations across MEA platforms.

	Data Processing	Strengths	Limitations	Ref.
HD-MEAs	- Spike sorting to single units - LFP and burst analysis - Functional connectivity analysis using cross-correlograms and transfer entropy.	- Enables long-term recordings from brain organoid slices with single-unit spike sorting - Axonal conduction velocity measurements - Quantitative analysis of functional connectivity.	- Access is restricted to networks near the slice surface rather than the full 3D volume of the organoid.	[19]
Mesh MEAs	- High-rate sampling - High-pass filtering - Noise-based threshold detection - Analysis of spontaneous burst patterns.	- Uses flexible mesh electrodes to minimize tissue damage - Allows long-term recording inside organoids.	- Provides relatively low electrode counts and spatial resolution compared with CMOS-based MEAs - Recordings are often limited to electrodes located near the basal surface.	[25]
Shell MEAs	- Threshold-based spike detection - Spike count - SNR evaluation.	- Wraps around spherical organoids - Increasing contact area and spike detection efficiency.	- Requires manual folding and organoid insertion - Limits throughput and can introduce user-to-user variability.	[27]
Kirigami MEAs	- Single-unit spike sorting - Long-term firing-rate tracking - Quantification of optogenetic - Pharmacological responses.	- Conforms to the shape of the organoid - Enables stable long-term attachment and recording.	- Involves complex pattern design and fabrication, resulting in a high operational barrier - Currently offers a limited number of channels.	[28]
Multifunctional 3D MEAs	- Spike sorting - Amplitude-threshold (≈3× noise) spike detection - Burst and synchrony/network analysis.	- Integrates multiple functions (e.g., electrical recording, stimulation, optical or chemical modulation) to enable detailed analysis of neural circuit dynamics.	- Requires customized packaging and complex fabrication, - Insertion-type structure may impose mechanical burden for long-term applications.	[32]

## Data Availability

No new data were created or analyzed in this study.
